# HIPTox—Hazard Identification Platform to Assess the Health Impacts from Indoor and Outdoor Air Pollutant Exposures, through Mechanistic Toxicology: A Single-Centre Double-Blind Human Exposure Trial Protocol

**DOI:** 10.3390/ijerph21030284

**Published:** 2024-02-29

**Authors:** Thomas Faherty, Huda Badri, Dawei Hu, Aristeidis Voliotis, Francis D. Pope, Ian Mudway, Jacky Smith, Gordon McFiggans

**Affiliations:** 1 School of Geography, Earth and Environmental Sciences, University of Birmingham, Edgbaston, Birmingham B15 2TT, UK; f.pope@bham.ac.uk; 2 Division of Infection, Immunity and Respiratory Medicine, University of Manchester, 2nd Floor Education and Research Centre, Wythenshawe Hospital, Southmoor Rd., Manchester M23 9LT, UK; huda.badri@manchester.ac.uk (H.B.); jacky.smith@manchester.ac.uk (J.S.); 3 Manchester University NHS Foundation Trust, Manchester M13 9WL, UK; 4 Centre for Atmospheric Sciences, Department of Earth and Environmental Science, School of Natural Sciences, University of Manchester, Manchester M13 9PL, UK; dawei.hu@manchester.ac.uk (D.H.); aristeidis.voliotis@manchester.ac.uk (A.V.); g.mcfiggans@manchester.ac.uk (G.M.); 5 National Centre for Atmospheric Science, Department of Earth and Environmental Science, University of Manchester, Manchester M13 9PL, UK; 6 MRC Centre for Environment and Health, Imperial College London, London W12 0BZ, UK; i.mudway@imperial.ac.uk; 7 NIHR Health Protection Research Unit in Environmental Exposures and Health, Imperial College London, London W12 0BZ, UK; 8 NIHR Health Protection Research Unit in Chemical and Radiation Threats and Hazards, Imperial College London, London W12 0BZ, UK

**Keywords:** air pollution, cognitive function, dementia, indoor air, human exposure, study protocol

## Abstract

Over the past decade, our understanding of the impact of air pollution on short- and long-term population health has advanced considerably, focusing on adverse effects on cardiovascular and respiratory systems. There is, however, increasing evidence that air pollution exposures affect cognitive function, particularly in susceptible groups. Our study seeks to assess and hazard rank the cognitive effects of prevalent indoor and outdoor pollutants through a single-centre investigation on the cognitive functioning of healthy human volunteers aged 50 and above with a familial predisposition to dementia. Participants will all undertake five sequential controlled exposures. The sources of the air pollution exposures are wood smoke, diesel exhaust, cleaning products, and cooking emissions, with clean air serving as the control. Pre- and post-exposure spirometry, nasal lavage, blood sampling, and cognitive assessments will be performed. Repeated testing pre and post exposure to controlled levels of pollutants will allow for the identification of acute changes in functioning as well as the detection of peripheral markers of neuroinflammation and neuronal toxicity. This comprehensive approach enables the identification of the most hazardous components in indoor and outdoor air pollutants and further understanding of the pathways contributing to neurodegenerative diseases. The results of this project have the potential to facilitate greater refinement in policy, emphasizing health-relevant pollutants and providing details to aid mitigation against pollutant-associated health risks.

## 1. Introduction

### 1.1. Background and Rationale

Air pollution is the primary global environmental risk to health that increases mortality worldwide [1]. Adverse effects of low-quality air on cardiovascular and respiratory systems are well-established [2,3], with our understanding of its effects on both short- and long-term population health advancing considerably over the last decade. There is also growing evidence that air pollution is neurotoxic [4,5], degrading the brain’s cellular structures [6], leading to neurocognitive decline [7] and delaying neurocognitive development [8]. However, there are still major gaps in our understanding of the most harmful components within the air we breathe and the mechanisms by which they induce adverse effects [9].

Lifetime exposure to low-quality air has been linked to greater risk of substantial cognitive deficits associated with progressive neurodegenerative diseases such as Alzheimer’s Disease [10], Parkinson’s Disease [11], and Multiple Sclerosis [12]. Chronic exposure to low-quality air is also associated with poorer than expected memory [13,14,15,16], psychomotor and sensory processing [17], and cognitive executive function [18,19] in clinically healthy children. Short-term episodes of exposure to low-quality air have also shown impact on these cognitive functions [20,21,22,23]. Evidence is, however, mixed with regards to the impact of certain pollutant mixtures on different cognitive domains [7]. It is therefore critical to assess a range of cognitive functions and changes as a function of differing common sources of air pollution.

Air pollution may manifest its acute and chronic impacts on the brain via direct interaction of inhaled particles, or desorbed chemical constituents, with non-neuronal glial cells and neurons in the brain, based either on their uptake via translocation along olfactory neurons to the olfactory bulb [24]; or their entry across the blood brain barrier from the circulation [25]. This has been supported by evidence of combustion-like nanoparticles in the brains of sentinel animals and humans from polluted environments [26], associated in autopsy samples with histopathological features of early dementia, microglia immune defence activation, and neuronal injury [27]. A second hypothesis proposes that the neurological damage reflects an indirect impact of air pollution induced systemic inflammation and its transmission to the brain across the glial-neurovascular unit [28,29]. There is therefore a clear necessity for this study to investigate evidence for both routes through both collection of blood to measure traditional toxicity endpoints, including pro-inflammatory biomarkers and markers for organ specific toxicity, and nasal lining fluids to identify the presence of neuronal injury markers.

We believe there is a strong need to respond to the emerging evidence of the impact of air pollution on the brain across the life course, from early life cognitive delays [14] to adverse mental health [30] and dementia risk. Moreover, the estimated cost of air pollution is GBP 20 billion per year in the UK, specifically attributable to cardiopulmonary mortality and morbidity alone [31]. The neurological effects carry significant economic and societal risks, above and beyond the cardiopulmonary risks, and will likely increase the economic and societal cost significantly. An enhanced understanding of which components of the air are drivers of these adverse neurological effects and the underlying causal mechanisms are, therefore, questions of fundamental importance to the health and economics of the nation.

The presented single-centre double-blind human exposure trial will investigate the effect of exposure to four common air pollutants deriving from cleaning products, diesel exhaust, cooking emissions, and wood smoke on cognitive function comparative to clear air. Pollutants from different sources display diverse physical and chemical properties, potentially resulting in a range of cognitive effects. For example, black carbon is the primary compound in particles emitted from engines and wood combustion during the flaming phase, often characterised by irregular shapes [32]. Conversely, organic compounds dominate aerosols from cleaning products [33], cooking emissions, and wood combustion during the smouldering phase, predominantly exhibiting spherical shapes [32,34]. Furthermore, the chemical composition and volatility of organic aerosols vary among sources, likely contributing to differences in cognitive effects. Therefore, the selected exposures not only contribute substantial fractions to indoor and outdoor air but also exhibit a range of physicochemical properties. The evidence for their influence on cognitive health is described in Section 2.4. The data generated from these human exposures represent an initial step in understanding the most hazardous components of outdoor and indoor air pollutants. This research aims to lay the groundwork for elucidating the causal pathways contributing to the development and exacerbation of neurodegenerative diseases. The results of this project will facilitate greater refinement in policy to place greater emphasis on health-relevant pollutants and provide these details to aid mitigation against pollutant-associated health risks.

### 1.2. Objectives

The primary objective of this study is to rank the acute effects of a range of air pollution exposures on cognitive function in healthy aged individuals with a family history of increased dementia risk.

The secondary objective of this study is to identify evidence of changes in inflammatory or immune signals post exposure to pollutants compared to clean air, exploring possible underlying mechanisms of any identified cognitive changes.

## 2. Methodology

### 2.1. Trial Design

**Figure 1:**
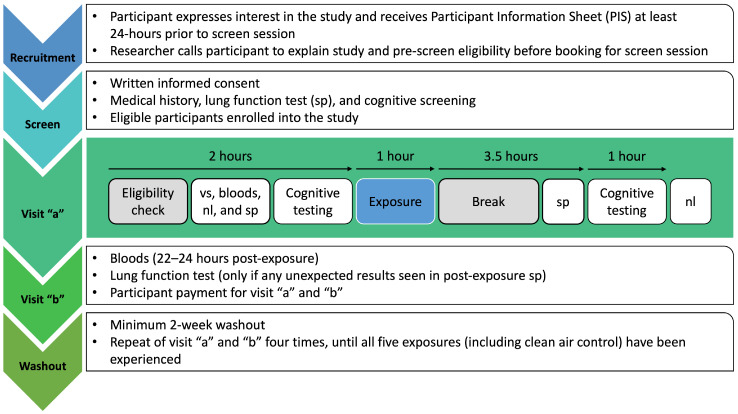
Study data collection procedure. vs. = vital signs; nl = nasal lavage; sp = spirometry (lung function).

This is a single-centre double-blind study of the effects of mixed air pollutants on the cognitive function of healthy human volunteers with a family history of increased risk of dementia. Participants will all undertake sequential controlled exposures to four air pollutants and clean air as control exposure, i.e., a repeated measures design. Cognitive testing, biological sampling, and physiological tests will be carried out before and after each exposure. The study design is outlined in Figure 1.

### 2.2. Study Setting

The HIPTox study will be carried out at the NIHR/Wellcome Trust Clinical Research Facility (MCRF), Grafton Street, Manchester and the Manchester Aerosol Chamber (MAC), Simon Building, University of Manchester, Manchester, UK.

### 2.3. Participant Volunteers

This study will aim to recruit 45 clinically healthy individuals aged 50 and above with a family history of dementia. See Appendix A for full inclusion and exclusion criteria. Recruitment began in May 2023 and due to significant challenges, as explained in detail in the discussion section, the originally planned sample size was reduced to 15 participants. The study is currently ongoing, and data collection is expected to be completed by the end of 2023. See Table 1 for study timeline.

#### 2.3.1. Power Calculation

Based on effect size and variance from a currently unpublished study [35] looking at cognitive load and attention (measured as change in response time ( Δ RT) after Diesel Exhaust exposure ( Δ RT = 22 ms, s.d. = 25 ms), a sample size of 30 subjects will have 90% power to detect significant response time changes of 15 ms assuming the standard significance level of *p* < 0.05. We acknowledge this current study is a different design utilising an older adult population and have, therefore, calculated for a slightly smaller effect size than that of the previous study to compensate for larger RT variance in older adults. As longitudinal studies are prone to extensive participant attrition, the sample size of 45 aims to allow full data to be collected for a minimum of 30 participants. Power calculations were performed using PS [36].

In response to recruitment challenges, we revised the sample size, prompting a re-exploration of the sample size estimate and power calculation to identify the minimum sample likely to yield valuable results. The proposed study design involves comparing the impact of four pollution exposures by examining the differences between pre- and post-exposure measurements relative to baseline measurements. The independent design from the previous study, featuring one measure per group, makes it unfeasible to extrapolate data. Therefore, under the assumption of a conservative correlation among repeated measures set at 0.5, a minimum sample size of 11 participants is needed to achieve 90% power in detecting a moderate (0.45) effect size at a significance level of 0.05.

#### 2.3.2. Patient and Public Involvement and Engagement (PPIE)

This project was supported by the Manchester University Foundation Trust’s public and patient engagement team (VOCALS) during the study conception and funding approval. Numerous public and patient engagement activities were carried out prior to protocol finalisation. These events helped shape the final study design including duration of the study. During the recruitment phase, we continued to engage with patients and the public via over 45 engagement events, including “Brain Health Day, Manchester”, local workshops, and pollution awareness events. We aim to carry out a post study completion engagement event with all the participants to disseminate the results alongside publication in scientific journals.

#### 2.3.3. Recruitment

Participants will be recruited from the general public. Volunteers who are employees of The University of Manchester or Manchester University NHS Foundation Trust will be permitted to take part if not involved with the project. Advertisement posters will be placed in public spaces including local community groups, places of worship, and NHS and university buildings. Social media advertisements will be run using official accounts of the University of Manchester, NHS Manchester Foundation Trust, and Research for the Future. We will also contact participants who have previously given permission to be contacted for research studies. Due to potential sex-dependent effects of aerosol exposure, we aim to collect equal numbers of biologically male and female participants.

To promote participant retention, participant payment will increase each visit so that there is an increasing incentive to continue participation as the study continues. Informal participant feedback will be collected each visit to improve subsequent visit comfort and amend the protocol as necessary. Data from those who discontinue participation, whether through personal withdrawal or change in eligibility, will be included in data analysis where inclusion of partial data is possible.

#### 2.3.4. Participant Blinding

This will be a double-blind study with both the trial participants and researchers/care staff responsible for data collection and analysis (MCRF staff; Dr Thomas Faherty; and Dr Huda Badri) blinded to the exposure. Other investigators will not be blinded as to the order of exposures. Participants will be informed about the five pollution exposures, but the sequence in which they encountered them will remain undisclosed. To assess potential unblinding among trial participants, the (unblinded) MAC team will ask participants to indicate which of the five exposures, including clean air, they believe they encountered following each one-hour exposure. Furthermore, participants will be asked to provide a confidence judgment along with their response. Trial participants and data collection staff will also be blinded during data analysis.

The PI will make the decision to un-blind the current participant exposure in the case of a serious adverse event (SAE) when knowledge of the chemical composition of the exposure is vital for appropriate clinical management or the participant’s well-being.

While there is a risk of unblinding due to smell, adding scents to mask pollutants or using external agents like menthol nasal rubs poses separate challenges, including potential alterations in the pollutant mixture and their impact on airway dilation, introducing additional confounding factors.

#### 2.3.5. Participant Safety and Anonymity

All participants enrolled in this study will be allocated a unique anonymous identifying code which will appear on all data collection documents. The hard-copy case report forms (hCRF) and source data files (SDF) will be kept within a locked room. All electronic data will be stored on secure password-protected servers with limited access to members of the research team.

Participant eligibility will be reviewed at the beginning of each visit (separated by a minimum two-week washout period) prior to air pollution exposure. Participation will be discontinued for those who do not match the eligibility criteria when reviewed.

The checking for the occurrence of adverse events (AEs) will begin from enrolment and will continue for the individual participant until their last visit. At each study visit, the researcher will assess eligibility and recent clinical history including the occurrence of adverse or serious adverse events (SAEs). Details of adverse and clinical events will be captured on the trial hCRF and eCRF. All SAEs will be recorded in the hospital notes, the eCRF, the hCRF, and the Sponsor’s SAE Recording Log. All SAEs will be reported to the Sponsor via the Research Office (RO) dedicated mailbox on an SAE form and the SAE Log will be sent to Sponsor on request. The PI will complete the Sponsor’s SAE form and the form will be sent within 24 hours of the Investigator becoming aware of the event. The PI will respond to any SAE queries raised by the Sponsor as soon as possible.

Expected Adverse Reactions are possible short-term respiratory symptoms such as cough/shortness of breath to susceptible individuals. We will minimise this risk by assessing the participants pre-enrolment onto the study for respiratory conditions. We will also conduct safety measures such as spirometry and exclude individuals with abnormal results which may also suggest a high risk to pollutant exposure. Investigators will complete the appropriate SAE form on the eCRF and an automatic email notification will be sent to the Trial Manager, PI and Sponsor.

A Suspected Unexpected Serious Adverse Reaction (SUSAR) is an adverse reaction that is classed as both serious and unexpected. The Trial Manager will ensure that SUSAR reports are un-blinded and reviewed by the PI or designee within 2 days and adjudicate whether the event constitutes a SUSAR. The Trial Manager will ensure that fatal or life-threatening SUSARs are reported to UREC as soon as possible, but no later than 7 calendar days after the receipt of the eSAE report. Any additional information will be reported within 8 days of sending the first report.

### 2.4. Exposures

The four major indoor (emissions from cooking and cleaning products) and outdoor pollutants (engine emission and wood combustion) emitted from the sources below have been selected as comparators to clean air as they are known to contribute substantial fractions to indoor and outdoor air.

Diesel exhaust: Previous studies demonstrate a clear negative effect on cardiopulmonary health [37,38] and there is evidence of neurological impacts of diesel exhaust in young healthy volunteers [39,40]; therefore, it is a pollutant of interest in this study;
Wood smoke: Wood smoke is a prevalent indoor and outdoor air quality issue in the UK [41]; in the last 10 years, its emissions have substantially offset the decrease in particulate matter (PM) from other sources, increasing by 33%, and it is likely to continue increasing in the medium-term [42]; therefore, identifying the impact on human cognitive health is of necessity at this critical juncture. The physical and chemical characteristics of the emissions from wood burning can vary widely depending on the fuel, burn condition, appliance, and dilution [43]. Here, we have selected a condition that enables us to produce stable and repeatable exposures for the participant group;
Cooking emissions: The significant contribution of cooking aerosol to indoor/urban air quality has recently emerged as an important topic within the aerosol community [34], but there is little solid evidence on its potential health impacts. A recent study did suggest that ultrafine aerosols from frying meat resulted in altered electrical brain activity in human subjects [44], warranting its further investigation here;
Cleaning products: Limonene, a household cleaning product ingredient, known for its citrus scent and effective dirt retention, is commonly found in indoor air at concentrations ranging from 20 to 50 μ g m^−3^ in residential buildings [45,46]. Peaks exceeding 70 μ g m^−3^ occur during cleaning episodes [47]. The presence of limonene and similar volatile organic compounds (VOCs) in indoor air raises health concerns, potentially causing sensory irritation, headaches, organ damage, or cancer [48]. Minimal evidence exists linking SOAs formed from household cleaning products to adverse cognitive effects. We have chosen limonene in the first instance to separate it from the other components across the multitude of cleaning products, whilst recognising its presence across lemon-fragranced products.

The study is designed to attempt to discriminate between the responses to exposure to 4 common pollutant types and a clean air control. The pollutant types have been selected as they make significant contributions to ambient air quality in indoor and outdoor situations. It is not possible to conduct exposures to emissions generated under all possible operating conditions of the individual sources. However, selection of a set of standard operating conditions for each source enabling the stable and repeatable generation of each pollutant emission type can enable comparison of the responses to exposure to each of the classes of pollutant. A description of the standard operating procedures and the chemical and physical characterisation of each source is the subject of a manuscript in preparation.

Participant exposures will be conducted at the Manchester Aerosol Chamber (MAC) facility. The MAC facility is part of the ATMO-ACCESS atmospheric simulation chamber network. It is an 18 m^3^ fluorinated ethylene propylene Teflon bag housed in a temperature- and humidity-controlled enclosure and illuminated by a combination of wavelength-filtered arc and halogen lamps. Clean inlet air is ensured by scrubbing with a series of filters and conditioners. Particle and gas levels are controlled either by injection of individual components or by coupling to a variety of real pollutant sources [49]. This facility as well as the techniques and specific instruments employed here have been used in many previous chamber studies [50,51,52]. For each pollutant mixture, a full characterisation will be conducted to identify the exact chamber conditions and experimental setpoints to achieve target pollutant concentrations to within acceptable limit. All experimental setpoints are computer-controlled and continuously monitored, ensuring participant safety, and enabling repeatability.

The aim is to achieve desirable PM (particle matter) mass concentrations (150 μ g m^−3^) for human exposure studies. The chemical and physical characterisation of particle and gas phase pollutants from each source was conducted using online instruments, and a protocol has been developed for generating desirable and reproducible PM mass concentrations from each source. Detailed information on this process is the subject of a manuscript in preparation. A brief description is provided below.

Diesel exhaust: For the generation of diesel emissions, we selected a diesel generator (Manufacturer: Lister Petter) that can provide representative and safe levels of PM/NO_*X*_. The engine is run on idle for 5 min and then the emissions are transferred to the chamber until the desired PM concentration is reached;
Wood smoke: To replicate a representative wood combustion condition in the atmospheric environment, a commercial Ecodesign-ready wood-burning stove (Esse 175F; sd) is employed in this study. Wood samples are burned inside the stove, positioned outdoors adjacent to the laboratory building, and using ambient air. Exhaust emissions are sampled from a port in the 125 mm diameter flue and drawn into the chamber via an ejector diluter (Dekati^®^ eDiluter^™^ Pro; Dekati Ltd., Kangasala, Finland);
Cooking emissions: The cooking process is carried out in a cooking chamber (approximately 0.7 m^3^). The aim is not to replicate any particular recipe, but to generate combustion emissions that will be components of some recipes, but that will be distinct from wood burning or diesel exhaust. To reduce variability, ingredients are kept limited, using 100 mL of olive oil and 2 pieces of pork chops to generate particles under standard frying conditions using an induction hob at a single setpoint. Once pollutants have accumulated sufficiently within the cooking chamber, clean air from our chamber system is used to flush all cooking-related pollutants into the aerosol chamber;
Cleaning products: Since limonene is a major compound found in cleaning products, we utilize it as a representative compound to produce secondary organic aerosols (SOA). For this study, we employ 130 ppb of limonene and 50 ppb of O_3_ to generate particles. Under these conditions, all the O_3_ is consumed, resulting in a residual limonene concentration of approximately 40 ppb, well within the safe range.

**Figure 2:**
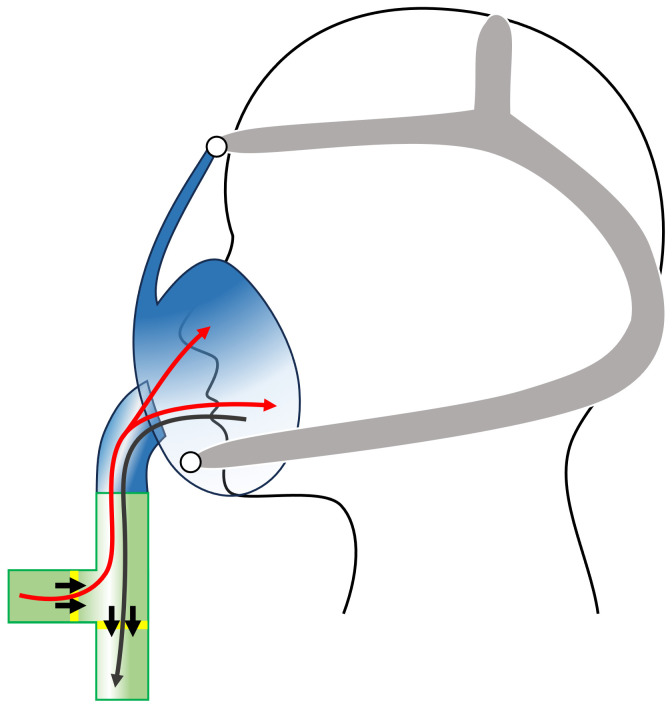
The two-way valve and mask set-up during exposure. Black arrows show airflow direction of one-way valves. Participants inhale air from the chamber (red line) and exhale into the surrounding room (dark grey line).

Participants will undertake a 60-minute exposure to a controlled concentration of common air pollutants in addition to a clean air exposure to act as a control. Participants will be fitted with a mask covering the nose and mouth (VariFit^™^ NIV non vented mask; Sleepnet Corporation, Hampton, NH, USA) attached to the aerosol chamber via a plastic pipe. The mask will use a two-way valve (Anti-pollution T-piece directional valve) allowing participants to inhale air from the chamber without forced air pressure and exhale into the surrounding room, avoiding contamination of the chamber air mixture. See Figure 2. As each participant completes pre- and post-exposure measures for each air mixture, including a clean air control, they serve as their own control. This approach helps mitigate possible individual differences in lung size and exposure uptake. Additionally, as participants are only included in the study with no underlying significant lung issues, and are at rest during exposure, it is unlikely there would be significant differences in tidal volume [53], further supporting the argument that variations in exposure due to breathing rate or body size are not a significant concern in the study.

A review of adverse event reporting from previous wood smoke and diesel exhaust exposure chamber studies utilising higher pollutant concentrations showed no evidence of adverse events. See Supplementary File S2 for full details.

### 2.5. Physiological Measures

#### 2.5.1. Spirometry

Spirometry will be a safety measure in this study. Volunteers will be asked to perform spirometry, to achieve 3 reproducible forced expiratory volume (FEV1) and forced vital capacity (FVC) measurements according to the American Thoracic Society/European Respiratory Society guidelines [54].

#### 2.5.2. Blood Tests

Blood will be taken as part of the assessment of inflammatory response. This will be plasma and RNA samples 5 mL (3 bottles pre exposure and 3 bottles on visit b), a total of 30 mL per participant per visit.

These collected samples will be centrifuged and transported to Imperial College Health Care NHS trust for analysis and storage. These will be stored for 2 years under their Human Tissue Authority License number 12,275.

Once analysis has been completed, any additional samples will be stored in biobanks for up to 30 years if participants have consented to this optional/additional section in the ICF. If a participant does not wish to have their samples stored in a biobank, this will not affect their participation in the study. It is expected that these biobanked samples will be accessible to researchers for further analysis in the future.

We intend to complete analyses for the following effects. Systemic inflammation will be assessed by measuring plasma concentrations of TNF- α , IL6, IL8, and C-reactive protein (CRP). BBB disruption, neuroinflammation, and neuronal injury will be assessed by determination of S100 calcium-binding protein B (S100B), Glial fibrillary acidic protein (GFAP), and Neurofilament light chain (NF-L), respectively, using commercial ELISAs.

#### 2.5.3. Nasal Lavage

Participants will undertake nasal lavage with 15 ml pre-warmed normal saline solution (0.9% NaCl). Sterile solution will be instilled into the participant’s nose whilst their head is flexed and chin towards their chest. There will be a total of 10 sprays in one nostril, whilst the other is closed off. This will be repeated four times and then, using a glass beaker, the fluid is collected. The whole process is then repeated with the other nostril for a total of 50 sprays in each nostril. The sample is then decanted into a centrifuge tube and centrifuged for 10 mins at 800× *g* at 4 °C.

We will employ this lavage method to ensure the collection of pure samples from the nasal lining fluid, enabling the investigation of damage-associated molecular patterns (DAMPs) as an early signal driving downstream immune response.

The nasal lavage samples will then be transported to Imperial College Health Care NHS trust for analysis and storage. These will be stored for 2 years under their Human Tissue Authority License number 12,275. These samples will then be destroyed as per the terms of the license.

We intend to complete analyses for the following effects. Cell free lavage will be analysed for markers of acute neutrophilic inflammation (IL-6, IL-8) and activation (Myeloperoxidase, lactoferrin) to provide an in vivo marker of the capacity of each of the aerosols to induce upper airway inflammation, paralleling an approach adopted previously [55]. In addition, lavage fluid antioxidant concentrations, ascorbate urate and the GSH/GSSG ratio, will be determined according to established assays [56].

### 2.6. Cognitive Measures

#### 2.6.1. Dementia Assessment

The General Practitioner Assessment of Cognition [57] (GPCog) is a screening tool for global cognitive impairment seen in those with dementia. The Participant Examination assessment (see Supplementary File S3) will be completed as part of eligibility screening. Briefly, participants are given a name and address to immediately repeat and remember. Participants are then asked to provide the exact day, complete two diagrams highlighting clock time, and tell the experimenter something that has happened in the news in the past week. Participants then recall the name and address they were asked to remember at the start. Usually, a score of 8 or below would involve additional information (Step 2: Informant Interview) from an informant, i.e., a family member who would know the participant and their current functioning compared to previous years. As we will not have access to an informant, participants will be allowed one error, so a score of 8 or 9 will indicate that participants are cognitively intact and can continue with the study.

#### 2.6.2. Cognitive Tasks

For cognitive assessment, we will utilise five tasks—one standardised manual task and four internally designed computer tasks. Two of the computer tasks will integrate novel elements into previously used paradigms and two will adhere to more commonly used methodologies, ensuring alignment with established research. This approach combines standardised rigour with innovative, internally developed tasks, providing a comprehensive evaluation of cognitive domains. A summary of the five tests is detailed in Table 2 and further description is given below. The full methodology of each task is provided in the Supplementary Materials (File S4).

The Purdue Pegboard Test [58] (PPT) is a manual test not requiring a computer. The other four cognitive tasks will require a Windows 10 computer running Matrix Laboratory (MATLAB) version R2022a [59]. These four tasks are in the format of a MATLAB script utilising the Psychophysics Toolbox version 3.0.18 [60]. The five tasks are described in more detail below.

Spatial *n*-Back Task: A working memory task using spatial locations of stimuli. This is a measure of participant ability to hold multiple pieces of information in the brain at one time and recall that information when necessary;
Face Identification Task: A selective attention task using particularly distracting face stimuli. This is a measure of cognitive control, a facet of executive function, specifically, participant ability to ignore distracting stimuli and focus on task goals;
Purdue Pegboard Test: This is a standardised test to measure of motor control, specifically participant hand and finger dexterity to manoeuvre small objects;
Expression Recognition Task: A Go/No-Go task using affective facial stimuli. This is to measure socio-emotional cognition, specifically, participant ability to identify the emotion expression of a human face at speed;
Psychomotor Vigilance Task: A simple reaction time task. This measures both sustained attention, i.e., participant ability to focus their attention for a sustained period of time, and psychomotor speed, i.e., speed of their response to changes in the visual field.

### 2.7. Data Analysis

Data generated by this study will be analysed using longitudinal statistical modelling designed to account for the repeat measures in individual subjects (e.g., general estimating equation or random effects modelling, depending on the extent and nature of any missing or partial data). This will describe and account for changes in pre-exposure measures over time, comparing the effects of the different air pollution exposures to the clean air control.

Cognitive subscales will be entered into this process separately, to aid in pairing aerosols with undesirable changes identified in each cognitive domain. Our goal is to ensure an equal representation of biologically male and female participants, acknowledging potential sex-dependent effects of exposure. Consequently, biological sex will be entered as a covariate of interest during analysis.

In addition to ranking cognitive impacts in relation to the exposure sources, these data will be related to biomarkers of systemic and neuro-inflammation/injury to test the secondary hypothesis that acute cognitive effects and more severe downstream neurological impacts reflect underlying inflammatory processes.

Specifically, our primary and secondary end points are as follows:

Primary end point:

Evidence of impairment in cognitive function post exposure as measured by any of these cognitive tasks compared to clean air:

1. Difference in the Approach bias measured via the Expression Recognition Task post pollutant exposure;
2. Change in the Cognitive Control response time post pollutant exposure;
3. Change in the 2-back discrimination index post pollutant exposure.

Secondary end point:

1. Change in performance on any of these cognitive tasks post pollutant exposure compared to clean air:
  - Psychomotor Vigilance Task;
  - Purdue Pegboard Test;
2. Evidence of inflammatory or immune signals post exposure to pollutants compared to clean air;
3. Change in lung function post pollutant exposure compared to clean air;
4. Change in DNA post pollutant exposure compared to clean air.

### 2.8. Data Management and Monitoring

The study will be subject to the audit and monitoring regime of the University of Manchester. All research staff involved in this study will be familiar with the protocol and suitably qualified, trained, and competent to carry out the required techniques. Staff are also trained in ICH-GCP, local information governance policies, and the data protection act. The source documents will be defined as the printed reports for any study measurement completed on a piece of equipment with this capability, or the case report forms designed by and completed by research staff. All research related documentation including the site file, case report forms, etc., will be kept with the PI until the results of the study have been analysed and published. Then it will be boxed and archived offsite for a minimum of 5 years. Archiving and destruction logs will be generated.

## 3. Discussion

Using an integration of cognitive psychology, atmospheric sciences, clinical experimental medicine, and clinical biochemistry, this project aims to assess the comparative impact of source-specific aerosols on physiological and cognitive functioning. The methodology involves repeated testing pre and post exposure to controlled levels of pollutants, enabling the identification of acute changes in functioning and the detection of peripheral markers of neuroinflammation and neuronal injury.

We initially aimed for 45 participants; however, various recruitment challenges emerged during the study, such as the extended study duration, time-intensive visits, and the eligibility of those in the specific cohort of participants required. Despite efforts to alleviate the burden on participants through protocol reviews and an extensive recruitment drive involving over 45 Patient and Public Involvement and Engagement (PPIE) interactions, it became apparent that recruiting 45 participants was not feasible.

Upon reassessment, the study team adjusted the target to a smaller sample size of 15, assuming a similar dropout rate (33%). This adjustment was made to still maintain sufficient data for reasonable statistical power. Despite this adaptation, we acknowledge that the reduction in statistical power increases the likelihood of not detecting a true effect, leading to a higher risk of committing a Type II error due to wider confidence intervals. Detecting small effects is also challenging due to queries associated with using a shorter exposure time and concentrations relative to previous studies. Similarly, a smaller sample size is more susceptible to random fluctuations, which are anticipated in cognitive functioning. However, the repeated-measures design and control of participant activities during data collection aim to reduce this as a confounding variable.

We believe that, despite the increased risk of errors, the study is worthwhile with this smaller sample size, especially considering the feasibility of recruiting clinically healthy participants over the age of 50 with a family history of dementia. Furthermore, the identification of statistically significant results, showing that different pollution exposures result in different cognitive endpoints, has the potential to inform and refine policy. Indeed, hazard ranking of common air pollutants for the emerging health risk of dementia will be compelling to government, policymakers, and regulators, as well as the medical community and the public, facilitated by public health agencies and other key stakeholders.

This study aims to demonstrate that our methodology can discriminate between the cognitive impairment effects of several major representative sources of air pollution. Future research will be able to build on the results of this study by introducing further parameter space, including but not limited to air pollution co-exposures and pollutants aged under atmospheric irradiation, and targeting wider participant groups. The initial outcomes from the study resulting from this protocol will allow future work to focus on the areas of cognitive dysfunction identified.

## 4. Administrative Information

### 4.1. Reporting Standards

The SPIRIT reporting guidelines were used to prepare this manuscript [61].

### 4.2. Trial Registration

Trial registered on ISRCTN registration number 85634746, accepted on 3 April 2023.

### 4.3. Protocol Version

Current protocol version 1.6, 23 February 2023.

### 4.4. Sponsor Information

Sponsor details:

The University of Manchester

Ms Lynne Macrae, Faculty Research Practice Governance Coordinator

Faculty of Biology, Medicine and Health, 5.012 Carys Bannister Building, University of Manchester, M13 9PL

Email: FBMHethics@manchester.ac.uk

Telephone: 0161 275 5436

Roles and responsibilities of the sponsor:

The study sponsor has overseen the design of the study and will have oversight of the trial. The sponsor has ensured that the trial protocol, Participant Information Sheet (PIS), Informed Consent Form (ICF), General Practitioner (GP) letter, and submitted supporting documents have been approved by the University of Manchester Research Ethics Committee (UREC) and the Health Research Authority (HRA) prior to any participant recruitment taking place. This study will be conducted in compliance with the protocol approved by UREC and according to good clinical practice (GCP) standards and UK Clinical Trials Regulation.

The trial protocol, PIS, ICF, GP letter, and submitted supporting documents were approved by the HRA and UREC before participant recruitment. All subsequent substantial protocol amendments will be documented and submitted for ethical and regulatory approval prior to implementation. Site-Specific Assessment from the Trust Research & Development (R&D) and NHS R&D approval were granted prior to participant enrolment.

### 4.5. Trial Status

The current protocol attached is version 1.6, dated 23rd February 2023. Recruitment began in May 2023 and the study is currently ongoing. It is expected that data analysis will begin in January 2024.

### 4.6. Dissemination

Our expectation is that after data analysis, information from this study will be widely disseminated in the medical and scientific community.

## Figures and Tables

**Table 1:** Study time schedule of enrolment, exposures, and visits. Exp = Exposure.

	Recruitment		Enrolment		Post-Enrolment						
			**First Wave (W1)**	**Second Wave (W2)**	**Exp 1 (W1)**	**Exp 2 (All)**	**Exp 3 (All)**	**Exp 1 (W2)**	**Exp 4 (All)**	**Exp 5 (All)**	**Remnant Exp(s)**
Start date		09-Jan-23	02-May-23	01-Jun-23	15-May-23	19-Jun-23	24-Jul-23	21-Aug-23	11-Sep-23	16-Oct-23	20-Nov-23
End date		30-Jun-23	31-May-23	30-Jun-23	02-Jun-23	07-Jul-23	11-Aug-23	25-Aug-23	29-Sep-23	03-Nov-23	30-Nov-23
ENROLMENT											
Information Sheet		X									
Informed Consent			X	X							
Eligibility Screen			X	X	X	X	X	X	X	X	X
VISITS											
Exp 1	Visit A				X			X			X
	Visit B				X			X			X
Exp 2	Visit A					X					X
	Visit B					X					X
Exp 3	Visit A						X				X
	Visit B						X				X
Exp 4	Visit A								X		X
	Visit B								X		X
Exp 5	Visit A									X	X
	Visit B									X	X
MEASURES											
Pre-Exposure					X	X	X	X	X	X	X
Post-Exposure					X	X	X	X	X	X	X

**Table 2:** Summary of the cognitive tasks completed by participants pre and post exposure. Tasks will be administered in table order, starting with the Spatial *n*-back Task (SNB).

Task	Outcome Metrics	Cognitive Domain	Duration
Spatial *n*-back Task (SNB)	Working memory ability	Learning and memory	10 min
Face Identification Task (FIT)	Distractor suppression	Executive function	15 min
	Cognitive control		
Purdue Pegboard Test (PPT)	Gross movements of arms, hands, and fingers	Perceptual-motor function	10 min
	Fine motor extremity		
Expression Recognition Task (ERT)	Expression Discrimination	Socio-emotional cognition	15 min
	Approach bias		
Psychomotor Vigilance Task (PVT)	Sustained attention/vigilance	Attention	10 min
	Psychomotor speed		

## Data Availability

There are currently no plans to share participant-level datasets.

## Supplementary Materials

The following supporting information can be downloaded at: www.mdpi.com/xxx/s1, S1: Informed Consent Form; S2: GPCog; S3: Chamber Study Safety; S4: Cognitive Task Methodology; S5: Spirit Checklist. References in Supplementary Materials files have been cited as [62,63,64,65,66,67,68,69,70,71,72,73,74,75,76,77,78,79,80,81,82,83,84,85,86,87,88].

## Abbreviations

The following abbreviations are used in this manuscript:

eCRF	electronic case report form
ERT	Expression Recognition Task
FIT	Face Identification Task
GCP	good clinical practice
GPCog	general practitioner assessment of cognition
hCRF	hard-copy case report form
HRA	Health Research Authority
ICF	informed consent form
IRAS	Integrated Research Application System
MAC	Manchester Aerosol Chamber
MCRF	Manchester Clinical Research Facility
nl	nasal lavage
PI	principal investigator
PIS	participant information sheet
PM	particulate matter less than 2.5 micrometres in diameter
PPIE	Patient and Public Involvement and Engagement
PPT	Purdue Pegboard Test
PVT	Psychomotor Vigilance Task
RT	response time
SAE	serious adverse event
SDF	source data file
SNB	Spatial *n*-back Task
SOA	secondary organic aerosol
sp	spirometry
UREC	University of Manchester Research Ethics Committee
vs	vital signs

## Appendix A. Inclusion and Exclusion Criteria

The study inclusion criteria are:

- Age ≥ 50 years of age;
- Family history of dementia (first- or second-degree relative);
- Provision of signed, written, and dated informed consent, prior to any study specific procedures.

Participants will be excluded if any of the following criteria apply:

- Neurological or psychiatric disorder;
- General Practitioner Assessment of Cognition Score of ≤ 7;
- Current smoker (including e-cigarettes) or ex-smoker of less than 6 months abstinence, and < 20-pack/year history;
- Current pregnancy;
- Significantly abnormal spirometry;
- History of cardiac disease;
- History of chronic respiratory or airways disease;
- History of inflammatory diseases (e.g., rheumatoid arthritis, chronic periodontitis, inflammatory bowel disease);
- History of neurological or psychiatric disorders;
- History of organ transplant;
- Consumption of >3 units of alcohol in the 24 h preceding testing visit;
- Any vaccination in the week prior to the testing visit;
- Cold or flu in the 48 h prior to the testing visit;
- Visual impairment not correctable with glasses/contact lenses;
- Significant claustrophobia preventing the participant from wearing a mask over the nose and mouth for an hour;
- Concomitant medication use of neurologically active drugs such as opiates/ benzodiazepines/anti-epileptics, etc.;
- Substance misuse.
