# Supplementary material for: HIPTox—Hazard Identification Platform to Assess the Health Impacts from Indoor and Outdoor Air Pollutant Exposures, through Mechanistic Toxicology: A Single-Centre Double-Blind Human Exposure Trial Protocol
Source: Int J Environ Res Public Health. 2024 Feb 29;21(3):0. doi: 10.3390/ijerph21030284 (PMC11154498; doi:10.3390/ijerph21030284)
Supplement: Supplementary file 1 [file ijerph-21-00284-s001.zip › ijerph-2807268-supplementary/Sup2_GPCog.pdf]

Patient name: ____________________________

Testing date: ____________________________

GPCOG

STEP 1 – PATIENT EXAMINATION

Unless specified, each question should only be asked once.

Name and address for subsequent recall test

I am going to give you a name and address. After I have said it, I want you to repeat it. Remember this name and address because I am going to ask you to tell it to me again in a few minutes: John Brown, 42 West Street, Kensington. (Allow a maximum of 4 attempts.)

	Correct	Incorrect
**Time orientation**		
1. *What is the date?* (exact only)	☐	☐
**Clock drawing** (use blank page)		
2. *Please mark in all the numbers to indicate the hours of a clock.* (correct spacing required)	☐	☐
3. *Please mark in hands to show 10 minutes past eleven o'clock.* (11.10)	☐	☐
**Information**		
4. *Can you tell me something that happened in the news recently?* (Recently = in the last week. If a general answer is given, e.g. "war", "lot of rain", ask for details. Only specific answer scores.)	☐	☐
**Recall**		
5. *What was the name and address I asked you to remember?*		
John	☐	☐
Brown	☐	☐
42	☐	☐
West (St)	☐	☐
Kensington	☐	☐

Add the number of items answered correctly: **Total score:** ☐ **out of 9**

**9**	**No significant cognitive impairment**<br>Further testing is not necessary
**5 – 8**	**More information required**<br>Proceed with informant interview in step 2 on next page
**0 – 4**	**Cognitive impairment is indicated**<br>Conduct standard investigations

© University of New South Wales as represented by the Dementia Collaborative Research Centre – Assessment and Better Care; Brodaty et al, *JAGS* 2002; 50:530-534
